# Integration of Shangshan culture into the STEAM curriculum and teaching: results of an interview-based study

**DOI:** 10.3389/fpsyg.2023.1251497

**Published:** 2023-12-06

**Authors:** Chen Qian, Jian-Hong Ye, Chaocan Zheng

**Affiliations:** ^1^Jinhua Polytechnic, Jinhua, China; ^2^Faculty of Education, Beijing Normal University, Beijing, China; ^3^National Institute of Vocational Education, Beijing Normal University, Beijing, China

**Keywords:** belief-action-outcome model, C-STEAM, packaging design, fine traditional Chinese culture, practical teaching, STEAM course, teaching reform

## Abstract

**Introduction:**

Interdisciplinary science, technology, engineering, arts, and mathematics (STEAM) courses are a popular trend in international education than can help inculcate creativity in students. Although STEAM courses have been widely promoted in China, they are generally unsustainable because they are merely imitations of European and American courses and lack Chinese humanistic factors; a close integration between disciplinary ideas and thinking levels is also lacking. C-STEAM, which is designed to pass down China’s culture, is a form of STEAM education with local Chinese characteristics that are focused on integrating interdisciplinary knowledge with the thought process oriented toward cultural heritage.

**Methods:**

In this study, an innovative higher vocational college course with C-STEAM interdisciplinary principles was constructed, with art and design as the framework, and with the integration of Chinese local culture. Semi-structured interviews were conducted to sample 12 learners from a total of 90 students in the experimental class of the C-STEAM course. The study aimed to provide a deeper understanding of the effectiveness of taking a Packaging Design course combined with C-STEAM from multiple perspectives. After the interviews, based on the BAO model, coding statistics and thematic analysis were conducted to understand the learners’ beliefs, actions, and outcomes after taking the course, and their plans for acquiring C-STEAM interdisciplinary knowledge and learning Chinese local culture.

**Results:**

The integration of the Shangshan culture (上山文化) into the Packaging Design course proved the importance and significance of adding C-STEAM to the art design course, which helped us understand the specific feelings of students after completing the course and gain a deeper understanding of the changes in their knowledge and skills and their learning effects.

**Discussion:**

Integrating C-STEAM education into courses related to art and design is highly warranted to encourage students to apply their interdisciplinary knowledge to artistic exploration and creation. Moreover, to effectively develop a curriculum system with local characteristics, teachers should provide more opportunities for students to explore and learn C-STEAM in the future, and integrate multiple elements into their teaching. In general, a cultural perspective-based interdisciplinary education helps facilitate the creative transformation of traditional Chinese culture.

## 1 Introduction

STEAM (Science, Technology, Engineering, Art, and Mathematics) is an interdisciplinary integrated form of education (Dinh, 2021; Hacıoğlu and Suiçmez, 2022), focusing on heuristic teaching and learning (Perignat and Katz-Buonincontro, 2019; Sha et al., 2021) and guiding students in hands-on operation and critical thinking (Yakman and Lee, 2012; Fan and Ye, 2022), as well as aiming to improve their reading comprehension (Henderika, 2021; Shinya and Tomohiro, 2023), imagination, and creativity, which can truly spur the social change caused by innovation (Yakman and Lee, 2012; Quigley and Herro, 2016). STEAM education is not just limited to the fields of engineering and science (Hernandez et al., 2014; Honoka et al., 2022), but is also closely related to the education of physical applications of art and design (Fan and Ye, 2020; Jazariyah, 2023). Art and design are activities that involve solving new problems in different ways and creating new products (He et al., 2019), and require cognitive abilities of creative expression and appreciation (Qian et al., 2022). Solving tedious problems requires innovation and skills. An art and design course that develops creativity and fosters scientific awareness can improve students’ creative expression abilities (Hong et al., 2022), thinking skills, and techniques (Fan and Ye, 2020). In addition, quality-oriented education should run throughout the education system and be given special attention in both general and vocational education. Higher vocational education, in particular, is closely linked to the national economy (Chen, 2023). Only by providing better quality-oriented education for vocational education students can we help them find employment and, as a result, build a buoyant, robust national economy (Ye et al., 2023).

In recent years, STEAM education models have combined courses and physical design (Workosky and Willard, 2015; Tian et al., 2023). Such training can not only help students enhance their abilities to express themselves creatively and use broad knowledge to solve real-life problems (Jia et al., 2021; Shibata, 2022), but can also shape them into well-rounded talents with a distinctive innovative spirit (Daugherty, 2013; Yao and Liu, 2023). Many educators have emphasized that the reason for integrating STEAM education into art and design courses (Hong et al., 2019; Qian et al., 2022) is that designing products, such as textiles, packaging, and fashion products, involves different cognitive skills such as creativity, imagination, and other complex and multifaceted activities (Hong et al., 2019). Packaging design is a case in point. Its process is rigorous and complex, and requires consideration of the details of the material and design structure of packages, which is closely aligned with STEAM (Qian et al., 2022). Therefore, art and design courses are highly relevant to STEAM (Fan and Ye, 2020).

In the history of STEAM courses in China, studies have mostly focused on innovation in basic education, learning projects, and core literacy, while paying scant attention to higher education, especially higher vocational education (Qian et al., 2022). Meanwhile, overly imitating Western STEAM courses (Zhao and Fan, 2019) has resulted in inadequate humanistic thoughts (Zhan et al., 2023), a lack of integration with existing courses, and unsustainable development (Qian et al., 2022). Therefore, Zhan et al. (2022) suggested that we should consider China’s actual conditions to promote STEAM education integrated with excellent local culture (abbreviated as C-STEAM). This approach has provided a new direction for the localized development of STEAM education (Wu et al., 2022; Zhan et al., 2023), creating a long-term impact on training talents with local characteristics (Deng et al., 2022) and innovating China’s outstanding local culture education (Zhan et al., 2020; Yu et al., 2022).

Chinese local culture is a source of spirituality to the people of the nation that combines the values and ethics generally accepted by the native people. It embodies the profound ideology, artistic values, and esthetics of the nation’s culture, and serves as inspiration for creative expression (Huo et al., 2020; Zhao et al., 2021). Shangshan culture represents a Neolithic cultural site in Zhejiang province, eastern China, that exemplifies China’s local culture and has a strong influence across the world.

“Wannian Shangshan” is considered the origin of the world’s painted pottery civilization, Chinese farming villages, and the world’s rice cultivation culture (Jiang, 2013; Qian, 2019). Pottery, stone tools, wooden buildings, seed base, and various types of household equipment and production tools have been found at the Shangshan sites (Jiang, 2007). Ancient Chinese people who lived at these sites fully demonstrated diligence and wisdom through their scientific site selection, rice cultivation, construction of houses, decoration of pottery and stone tools, and measurement of tool making. The characteristics and cultural experiences of Shangshan culture are consistent with C-STEAM education’s science-based, reality-oriented, multidisciplinary knowledge and multisensory experiences that unite students (Qian et al., 2022). Therefore, integrating Shangshan culture into the STEAM course, students were instructed to design product packaging with the Shangshan culture and local specialties around the Shangshan sites as the theme. This could help them enhance their understanding and identity of national culture; develop their hands-on and creative expression skills through planning, research, observation, extraction of cultural elements, problem identification, practical work, and reflection; and apply their knowledge to solve real-life problems.

Therefore, the purpose of our study was to explore the similarities among traditional Chinese culture, STEAM education, and art design courses in vocational colleges, examine the characteristics of the art creation process, and further analyze the packaging design STEAM course based on the Shangshan culture, so as to construct a C-STEAM course model for art design. Semi-structured interviews were conducted with students with different levels of thematic design works, and finally, thematic analysis was conducted using the theoretical framework of the belief-action-outcome (BAO) model. Specifically, the present study aimed to identify Chinese college students’ feelings about taking the art and design course combined with C-STEAM. The results can not only help develop teaching practices with Chinese Characteristics in higher education and train art talents, but will also be helpful in protecting, utilizing, inheriting, and developing local culture, ultimately creating a win-win situation. At the same time, the results can provide theoretical support for the academic community from the perspective of art and design, and can make further contributions to diversifying the elements of disciplines.

## 2 Literature review

### 2.1 STEM education and art and design education

STEM education is an integrated form of education combining multiple disciplines (Fan and Ye, 2020). It intends to disrupt the boundaries between subjects and respond to how to cultivate students’ innovative thinking and problem-solving abilities (Shibata, 2022). Quigley and Herro (2016) reported that through STEM courses, students can become more engaged. These courses would make the curriculum more dynamic and interesting, thus stimulating students’ interest in learning and their desire to explore questions. During the courses, students build on a foundation of mathematical logic and use hands-on creation and artistic forms to demonstrate the essence of science and technology, thereby developing an ability to face challenges.

Art and design education, on the other hand, focuses on esthetic education and the cultivation of comprehensive qualities in designers (Schat et al., 2023). This includes cultivation of appreciative, expressive, and creative abilities (Peng, 2022). Art and design is a highly integrated discipline (Fang et al., 2022). For example, the packaging design process involves considering various factors such as packaging materials, size, structure, manufacturing techniques, and visual esthetics (Qian et al., 2022). Therefore, in the packaging design field, the emphasis on materials, functionality, structure, esthetics, and precise dimensions all closely align with STEM principles (Fan and Ye, 2020). Consequently, this study explored the integration of STEM in the packaging design context, thereby developing it into a learning curriculum activity and investigating students’ learning outcomes.

### 2.2 Curriculum design under the C-STEAM concept

C-STEAM implements localized interdisciplinary integration within STEM education (Zhan et al., 2022), primarily focusing on cultivating the ability of students to solve complex real-world problems through interdisciplinary thinking (Qian et al., 2022). It emphasizes inherently integrating interdisciplinary knowledge and thinking guided by the preservation of local cultural heritage (Zhan et al., 2023), rather than simply incorporating traditional culture into existing STEM courses (Huo et al., 2020; Zhan et al., 2022). C-STEAM guides students in understanding and appreciating local culture, conducting research, and incorporating scientific, technological, engineering, artistic, and mathematical knowledge into culturally enriched creative work (Zhan et al., 2022). This approach intends to bridge the gap between art, culture, and daily life, thereby nurturing students’ humanistic spirit, reinforcing their understanding and identification with national culture, and thus enhancing national pride and confidence (Qian and Wang, 2022). Moreover, art and design education based on Chinese cultural values facilitates the construction of an indigenous knowledge system in China. This system is a crucial foundation for the country’s development. The C-STEAM curriculum model discussed in this study is a comprehensive program integrating interdisciplinary and cross-disciplinary knowledge, mainly focusing on promoting China’s rich local culture, particularly the Shangshan culture, as a core value in the localized STEM education. This approach enables students to creatively apply knowledge from multiple disciplines in thematic projects, and facilitates the creative transformation and innovative development of China’s outstanding traditional culture. This, in turn, continually improves the country’s cultural soft power and global cultural influence.

### 2.3 The belief-action-outcome model framework

The Belief-Action-Outcome (BAO) model consists of belief, action, and outcome. Belief is an expression of a self-perceived state that drives an individual to hold strong beliefs and plan how to carry out actions to achieve a desired outcome; action is the process of achieving their goal; and outcome is the result of their actions (Pilditch and Custers, 2018). The BAO model has been considered effective in terms of explaining an individual’s action, their final outcome (Melville, 2010), and how their beliefs affect their subsequent action, which can have a significant impact on the outcome of their behaviors (Hong et al., 2022). In other words, the BAO model framework can help understand the cause of beliefs and explain how an individual’s beliefs influence the outcomes (Molla et al., 2014). Recent research has shown that the BAO framework can be helpful in explaining the relationship between learning beliefs, actions, and effectiveness in higher education (Nong et al., 2023). As noted above, the BAO model framework effectively explains the learning experience of learners. Therefore, this study used the BAO framework to explain how the beliefs of the interviewees affected their actual behaviors, outcomes, and performances after the course.

## 3 Methods

### 3.1 Course design

In this study, the “Packaging Design” course combined with C-STEAM was constructed; the framework is shown in Appendix 1 in Supplementary material, and its outline is based on the “packaging design” textbook edited by Liu et al. (2021). The curriculum is categorized into four units and is closely related to the higher education curriculum. The modules combine STEAM education and Shangshan culture (Fan and Ye, 2020), evolve from the elementary to the profound level, and promote a high degree of integration of interdisciplinary knowledge and practical skills, focusing on training students with comprehensive qualities of culture, science, skills, and esthetics to encourage them to grow into highly qualified and skilled talents with knowledge, technical ability, artistic ability, and cultural taste. The duration of the teaching experiment was 9 weeks (72 h-long periods in total). The students’ learning progress was monitored every lesson or every week by acquiring their design works, and they were instructed in a timely manner; expertise and skills in each field of C-STEAM were added by explaining each step so that students could fully understand the principles of packaging design and production (see Appendix 2 in Supplementary material).

**Figure 1:**
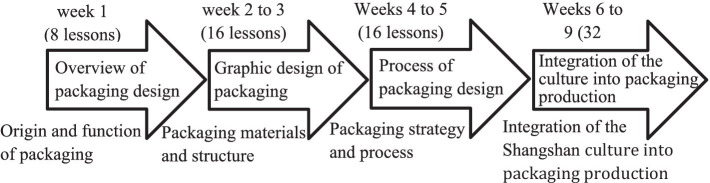
Curriculum planning chart.

The C-STEAM concept was used to develop the professional knowledge and skills of the Packaging Design curriculum, and was first developed by the lead teacher, subsequently examined by the course leader and the dean of the college, and finally reviewed and corrected by three experts and scholars with senior titles and with nearly 20 years of teaching experience. The integration of C-STEAM into packaging design was proposed to test the units of the Packaging Design course and the professional knowledge and skills required by students (see Figure 1).

### 3.2 Research method

Semi-structured interviews were conducted to learn about the students’ performance in the art and design course combining the C-STEAM concept. A total of 12 learners (who were at the high, intermediate, and low levels) were selected when they completed the course. Among them, four learners achieved excellent packaging performance, four learners achieved medium performance, and four learners delivered poor performance. At the end of the course, a 50–60 min interview was conducted with the learners to investigate their perceptions of the learning method and their acceptance of the curriculum change, including the cause of difficulties and what sparked their interest, to verify the results based on causal reasoning.

### 3.3 Research subjects

In this study, interest sampling was performed to select sophomore students in a higher vocational college in China majoring in art and design. After the classes were scheduled by the teaching office of the college at the beginning of the semester, 12 learners were selected from 90 students in the experimental class of the C-STEAM course who were at the high, intermediate, and low levels (Table 1). The sample comprised seven (58.33%) female students and five (41.67%) male students. A semi-structured interview was conducted with them at the end of the course, which was consistent with the purpose of this study.

**Table 1:** Basic information about the participants.

Number	Gender	Age	Awards	Previous majors
A	Female	21	National level	Art and design
B	Male	21	Provincial level	Art and design
C	Female	z	National level	Art and design
D	Male	21	College level	Digital media art and design
E	Female	21	None	Digital media art and design
F	Male	20	Provincial level	Art and design
G	Female	21	National level	Digital media art and design
H	Male	21	None	Art and design
I	Male	21	National level	Digital media art and design
J	Female	21	National level	Art and design
K	Female	21	Provincial level	Art and design
J	Female	20	None	Art and design

### 3.4 Research tools

Interviews were conducted according to the interview guide or presentation (which was used as a framework prior to the start of the interview) proposed by Lin et al. (2005). No severe constraint was imposed on the wording and sequence of questions, and the content was consistent with the research questions. Questions were asked in a flexible manner. In this study, the interview outlines were adapted according to the opinions of Zhan et al. (2022, 2023), and the specific questions are presented in Table 2.

**Table 2:** Interview outline.

Number	Questions
Q1	What did you like the most about the Packaging Design course integrated with C-STEAM?
Q2	What did you dislike the most about the Packaging Design course integrated with C-STEAM?
Q3	What difficulties did you encounter in this course? What methods did you use to overcome them?
Q4	What are the advantages (benefits) of the learning method in this course compared with conventional teaching (teachers lecture all the time)?
Q5	What are the advantages (benefits) of the learning style in this course compared with the conventional course design?
Q6	What is the biggest gain that the learning style in this course has brought you?
Q7	How did you learn about local culture, conventional Chinese culture, and Shangshan culture in this course? Could you integrate the culture into the creation of art and design and make use of it?
Q8	Did the use of interdisciplinary knowledge (culture, science, technology, engineering, art, and mathematics) help you to create “personalized” packaging? What did you find difficult?
Q9	Would you like to learn more in the C-STEAM course if you have an opportunity in the future?
Q10	Would you like to learn about traditional culture in the future (can you explain in detail what kind of traditional culture), and would you like to contribute to the transmission of China’s excellent local culture?
Q11	Will you rely on the culture in your future designs?
Q12	What would you recommend if you continue to take this course?

### 3.5 Data collection and analysis

First, written consent was obtained from the respondents, and the interviews were recorded using notes and audio recordings, ensuring that the information obtained was accurate. Immediately after the interviews, the respondents’ comments and opinions were sorted, and the responses that were consistent with the research topic were screened through a review form to establish authenticity and dependability (Wang et al., 2022). Furthermore, while reviewing the data in cooperation with a supervising professor to clarify the concepts and ensure the correctness and reliability of the analysis, three colleagues majoring in art and a part-time teacher from a company were invited to assist in data review and feedback because triangulation can avoid biases and distortions in data analysis caused by personal emotions or opinions.

A 50–60 min interview was conducted one on one with the respondents, which involved questions in Chinese on the Tencent meeting online platform. To ensure the effectiveness of the interview data, the data were immediately recorded and used to perform a micro-analysis (Strauss and Corbin, 1998). After the interviews, the data were coded using MAXQDA 2020.

After collecting the raw data, researchers sorted the data and conducted subsequent analysis through cross-sectional and longitudinal comparisons to understand the learning effects of the curriculum. A coding system was created, as shown in Table 3.

**Table 3:** Table of the data code.

Description	Code	Example	Description of the example
Subject	CN	C1N-33	33rd Student in class 1
		C2N-33	33rd student in class 2
Learning outcome	Learning	Learning C1N-33	Learning outcomes of the 33rd student in class 1 on March 2, 2023
records of students interviewed	interview	Interview F-01-Q01	Subject interviews
	F, M		Gender codes: female (F), male (M)
	1–12		Different interviewee codes
	Q1-Q12		Question numbers of the interview outline

## 4 Findings and discussion

The respondents’ words were transcribed, and based on the BAO model, subject analysis was performed to obtain relevant information. The learners shared their positive and negative learning experiences, their learning beliefs, actions, and outcomes, and specific reasons for participating in the C-STEAM Packaging Design curriculum, and provided suggestions for the course. Interview data were analyzed, and the results are shown in Table 4.

**Table 4:** Coding statistics.

BAO	Code list		Sub-category	Frequency
Belief	Positive	Interest in learning	Studying Chinese culture is extremely interesting	12
	Negative	Learning difficulties	Lack of knowledge of packaging	5
			Lack of creative thinking in the early stage of the design	10
			Superficial understanding of cultural meanings	7
Action	Positive	Interest in learning	Producing works of art is enjoyable	3
			Handcrafting is enjoyable Learning effect	4
	Negative	Learning difficulties	Difficulty in choosing packaging materials and structure	5
	Suggestions	Suggestions for improvement	Detailed explanations of each field of knowledge	10
			More kinds of teaching methods	3
		Expanded suggestions	Various local cultures should be integrated	3
			Study trips and design cases at home and abroad are expected to increase	4
			Interaction between teachers and students should be strengthened	6
Outcome	Positive	Interest in learning	Acquiring new knowledge of packaging is extremely interesting	4
		Learning effect	C-STEAM multidimensional learning has aroused interest	6
			Familiarity with new knowledge of packaging	6
			Ability to appreciate and learn from good design cases has improved	4
			Humanistic literacy and cultural confidence have improved	13
			Self-exploration and thinking ability have improved	8
			Problem-solving ability has improved	4
			Hands-on ability and production level have improved	3
			Ability to express creativity has improved	7
			Integrated design ability has increased	8
	Negative	Learning difficulties	Limited hands-on ability or production level	9
			Weak in mathematical calculation	18
	Intention	Learning intention	Traditional culture is profound and fascinating	13
			Continued improvement of cultural heritage and development	14
			Personal cultural attainment continues to improve	7
			Personal artistic design level continues to improve	5
		Improvement of abilities	Constant creative inspirations	9
			Comprehensive thinking of the functions of packaging	4
			Completion of works continues to improve	12
			Recognition of artwork continues to increase	3
			Packaging accuracy and protection continue to improve	6
		Cultural utility	Chinese national wisdom continues to be passed down	9
			Cultural identity and cultural confidence continue to grow	9
			Vitality of the artworks is lasting	10

### 4.1 Learning beliefs of the students taking the packaging design course combined with C-STEAM

The present study was conducted mainly to understand students’ experiences of taking an art and design course combining STEAM based on Shangshan culture. Based on the interview texts, the students’ learning beliefs can be divided into positive and negative aspects. Students’ positive learning experiences refer to their positive states such as favorable feelings, interest, gains, and learning outcomes of the C-STEAM integrated Packaging Design curriculum. Positive beliefs include a deeper interest in studying Chinese culture. In this study, students’ negative learning experiences refer to their negative perceptions of the Packaging Design curriculum combining C-STEAM, such as dislike, evasion, negation, repetition, and negativity. Negative beliefs include a lack of *a priori* knowledge of packaging, a lack of creative ideas at the early stage, and a limited understanding of the meaning of the culture.

Some participants found studying Chinese culture highly interesting (as shown below). They enjoyed learning about Shangshan culture, which in turn sparked their interest in the Packaging Design curriculum combining C-STEAM. The students considered Shangshan culture as the origin of Chinese culture and as having a long history and great cultural significance. The local Chinese culture is diverse and comprises the commonly accepted values and ethics of the Chinese people. Therefore, focusing on local Chinese culture is critical for promoting their long-term development.

I got a feeling of freshness and specialty when learning about the history and culture unique to Jinhua that I had never known before (M-03-Q01).

The respondents indicated their lack of knowledge of packaging (as shown below), lack of experience of packaging design before taking the Packaging Design curriculum, and their knowledge of using software for graphic design but not of 3D rendering. Due to their limited knowledge and skills, they tended to be emotionally affected, and disliked the course.

I didn’t know how to apply it [the culture] to a specific project before I learned about packaging because I had never come into contact with it before (F-01-Q02).

Some learners indicated that they lacked creative ideas at the start of the design. Specifically, they were confused about choosing a product for packaging, the structure to wrap products, and the painting style to decorate their packages. Furthermore, some respondents would follow the herd because most used the rice grain as the main image of “Shangshan Xiaobai (上山小白).”

The first step of choosing products based on the theme was the hardest part because I didn’t know what to choose (M-08-Q03).

After I overcame the difficulties encountered in this course, I came up with a new idea (F-05-Q03).

Some students believed that their understanding of the cultural meanings was superficial. Owing to the extensive and profound Shangshan culture, the subjects generally worried that their understanding of the culture was one-sided and that they could not completely grasp the cultural characteristics and meanings, indicating their lack of confidence in the extracted cultural elements and their design works.

I would make mistakes in the design, fearing that I could not capture the characteristics of the Shangshan culture and would stray from the point, not reflecting the culture (F-07-Q03).

### 4.2 The learning actions of the students taking the packaging design course combined with C-STEAM

Based on the interview texts, students’ learning actions can be divided into positive and negative aspects and learning suggestions. The positive aspect includes the joy of art creation and pleasure in producing works by hand, whereas the negative aspect includes the difficulty in choosing packaging materials and structures.

In this study, learning suggestions refer to the students’ suggestions or insights after taking the Packaging Design course combining C-STEAM. These suggestions can be useful for optimizing and developing the course in a better manner. It can be concluded that the suggestions for improvement include more detailed explanations of each area of knowledge and various teaching methods. Expanded suggestions include integrating various local cultures, increasing the number of study tours and domestic and international design cases, and promoting interaction between teachers and students. The reasons were comprehensively analyzed.

Some students found producing works of art enjoyable because creating things by combining the long history and culture with modern art not only made them happy and was enjoyable, but it also resonated with other people.

Combining the culture assigns a different cultural meaning to the design work and makes people understand deeper meanings (M-02-Q01).

Some participants enjoyed crafting, and they mentioned that they would practice crafts in their spare time, so they had experience of practicing crafts before taking the course. Furthermore, they stated that they would enjoy giving play to what they were good at and interested in during the course.

I excel at running software and drawing designs, so I like it (F-07-Q01).

Choosing packaging materials and structures was perceived to be difficult by some students (as shown below). This difficulty was attributed to the various packaging materials that are currently available, such as different types of paper, as well as the materials for making different products. Therefore, they tended to be bored with repeated choices. Furthermore, some respondents stated that various packaging structures are available, and choosing the right package was tedious. Therefore, they tended to be confused or annoyed because it required considerable time to experiment during the production process.

When I began designing structures, I could not find a suitable box. I tried out many different packaging forms to store the products, but I did not know whether they were suitable (F-12-Q02).

Some participants suggested detailed explanations of each area of knowledge (as shown below), especially mathematics. Most students felt that their mathematics skills were weak and that they required detailed explanations for accurately measuring the size and assembling the package. Typography skills were also deemed necessary. Though they had applied their previously acquired skills to graphic design, they were required to apply those skills to three-dimensional design, for which they required detailed explanations.

What we need to learn is a bit too comprehensive, and there is no detailed explanation of a particular area (F-11-Q05).

Some respondents felt the diversity of teaching (see below) and enjoyed greater freedom in the packaging design class. They suggested that teachers should focus on the students who lacked initiative; teach using a variety of approaches such as class discussions, group discussions among students, and interactive discussions between teachers and students; and provide frequent instructions on their homework to promote student development.

We enjoyed greater freedom in the class, so some students who lacked initiative would muddle along in the class (F-12-Q05).

Some students suggested that apart from Shangshan culture, other local cultures should be integrated (see below). They proposed that the integration of other traditional cultures, instead of only one culture, into the course is necessary because China is a large country with an exceptionally rich cultural heritage.

In terms of culture, the course should be more diverse to give us more choices (M-04-Q12).

Some participants suggested that more study trips and domestic and international design cases should be incorporated (as shown below). They believed that field trips would be more effective than learning indoors. Furthermore, they expected that more domestic and international art and design cases should be shared so that they can extend their knowledge and broaden their horizons.

It is better to share more creative cases, for example, western design cases or domestic award-winning cases (F-01-Q12).

If there is a chance, we can go and visit the Shangshan site, so that the course content is richer (M-02-Q12).

Some respondents suggested that the interaction between teachers and students should be strengthened because most students lacked relevant learning experience before opting to take the course. They tended to be confused about future courses, and some introverted students did not take the initiative to approach teachers for help. Therefore, they suggested that teachers should focus on such students and strengthen communication with them.

Sometimes students are too shy to take the initiative to communicate with their teachers. They should not always have their head buried in books. They need more communication with and assistance from their teachers (F-07-Q05).

### 4.3 The learning outcomes of the students taking the packaging design course combined with C-STEAM

According to the interview texts, the students’ learning outcomes can be grouped into positive and negative aspects. The positive aspect includes an interest in learning and academic performance, such as a greater interest in acquiring new knowledge of packaging, a growing interest in multi-dimensional learning of C-STEAM, a deeper understanding of new knowledge of packaging, greater abilities to appreciate and learn from excellent design cases, improved humanistic attainments, boosted cultural self-confidence, greater ability to explore and think independently, better problem-solving skills, greater manual dexterity, greater creativity, and greater ability to produce designs. The negative aspect includes poor manual dexterity and arithmetic skills.

Some respondents found acquiring knowledge of packaging to be very interesting (see below). Generally, students believed that the transformation of a flat sheet of paper into a three-dimensional box could help them feel different visual sensations, and learning how to run the “Baoxiaohe” software and measuring and calculating sizes expanded their knowledge and impressed them, making them believe that learning is fun.

It gives me a great sense of achievement to transform a piece of paper into a three-dimensional box by measuring each point, drawing lines, cutting, folding, and pasting (M-09-Q01).

Some participants were interested in learning C-STEAM (see below), especially multidimensional learning. In addition, if one or two knowledge nodes interested them, they tended to be interested in the whole course and pay more attention.

The course was taught in a non-traditional way, and aroused the students' curiosity and increased their attention (F-03-Q04).

Some students were familiar with packaging (as shown below). They could quickly grasp the whole design process, including market research, cultural integration, packaging material selection, designing structures, drawing unfolded and effect pictures, and printing, all of which brought them a sense of achievement.

Making an unfolding drawing on the computer, then drawing an effect picture, and finally printing to make the complete work brought me a sense of achievement and is my biggest gain in the packaging design class (M-04-Q06).

The participants’ confidence in their ability to appreciate and learn from excellent design cases increased (as shown below). By appreciating vivid and interesting packaging cases, especially anthropomorphic design works, they could not only broaden their horizons and improve their esthetics but also learn how to apply them to their studies.

The teacher would show us many interesting and award-winning packaging cases with anthropomorphic packaging designs in class (F-01-Q01).

All learners agreed that they had gained a deep understanding of humanities and increased their self-confidence in their own culture (as shown below). Students gathered at the school from all over the country to learn the traditional local culture, deeply experience the unique charm of various cultures, and produce their works of art combining the culture, which embedded the culture in their hearts and increased their self-confidence in their culture.

I have learned a lot about Jinhua culture and have come under the influence of the culture. Therefore, my self-confidence in our own culture has increased and I am proud of being Chinese (F-10-Q06).

Some respondents felt that their ability to explore and think independently had improved (see below). They mentioned that when designing packages, they not only explored the wisdom of the ancient texts, extracted cultural elements, and incorporated them into their designs, but also understood how to improve their ability to manage cost based on market demands.

I would not only consider its beauty or structure but also its economy and practicality (F-01-Q06).

Some students believed that their problem-solving ability had improved after the course. They mentioned that they tended to be confused about the design theme and specific direction in their works, but with the help of their teachers and through constant efforts to integrate culture, sharing cases, thinking independently, and studying online, their problem-solving ability had improved.

I could communicate well with my classmates and could communicate easily with my teachers when I ran into intractable problems (M-06-Q04).

The respondents felt their hands-on skills and production level had improved after the course (as shown below). They mentioned that the continuous processes of measurement and production increased their enthusiasm because packaging design requires calculating dimensions, drawing, and folding.

My manual skills have sharpened and my enthusiasm for making has grown, and I have learned how to do manual work better, such as calculating dimensions, making drawings accurately, and the knowledge of deciding which to use or discard (F-03-Q06).

Some participants felt that their creative expression ability had improved (see below). They believed that compared with previous designs, their thought processes changed after completion of the course. Specifically, the packaging structure could be extended outward, and the packaging decoration can be anthropomorphic, providing inspiration for their works.

The integration of local culture and packaging design provided us with more creative inspiration, allowed us to think fresh thoughts, feeling that there was more room for creativity (F-07-Q06).

Some students believed that their comprehensive design skills improved (as shown below) because the Packaging Design curriculum combined the knowledge of culture, science, technology, engineering, art, and mathematics with that of running various design software packages such as “Baoxiaohe” and AI. They mentioned that the course allowed theoretical knowledge to be closely integrated with practical creation, which strengthened their dynamic and active thinking and broadened their design horizons.

The knowledge I had acquired before was all about graphic design, but the knowledge I have gained in this class is about 3D design, which is up-to-date and full of freshness (M-09-Q01).

The respondents believed that their personal hands-on ability was limited and their production level was low (as shown below). Specifically, when making unfolded drawings and drawing folding boxes, they would often mix up the folding and cutting lines. Therefore, they tended to make repeated mistakes when cutting and had to draw and assemble again, leading them to become depressed and frustrated.

I often had to remake boxes because the folding and cutting lines were mixed up, or I bored the wrong folding lines or wrong holes, so I had to redraw and refold (F-10-Q02).

All participants felt that their ability to perform mathematical calculations was limited (see below), which further limited their ability to measure and calculate the package sizes. They obtained results that were either too long or too short, making complete assembly packaging difficult for them. Therefore, the more frequently the participants redid their work, the more likely they were to experience negative emotions.

It was difficult to calculate sizes. Being about 0.5 cm larger will result in a relatively empty box, which tends to cause waste and high costs (F-12-Q02).

### 4.4 Learning intentions of the students taking the packaging design course combining C-STEAM

In this study, intentional learning experience refers to students’ behavioral responses, such as aspirations, hopes, and intentions, after completing the Packaging Design course combining C-STEAM.

In terms of the students’ intentions after the course, this study concludes that the reasons for continued participation were the extensive, profound, and fascinating traditional culture; continued efforts to pass down and develop the traditional culture; and continuous improvements in people’s cultural attainments and art design level. Interdisciplinary knowledge includes creative inspiration, comprehensive thinking about the functions of packages, increased completion of works, continuous improvement of artwork recognition, and increased packaging accuracy and conservation. The utility of culture includes the continuous transmission of Chinese national wisdom and the enduring vitality of artworks. These results are comprehensively explained.

Some respondents believed that the traditional culture is extensive, profound, and fascinating (see below). They cited examples of traditional Chinese culture including the Dunhuang murals of the Tang Dynasty and Song Dynasty, the Tujia culture of Hunan Province, Chinese monochrome pictures, and the Shangshan culture of Zhejiang province. Deep in meaning and rich in content, Chinese culture is a spiritual home to all Chinese people.

I want to learn more traditional culture, like Chinese paintings. Since childhood, I have been very interested in landscape painting, which has a special pleasing quality (F-10-Q10).

All respondents were willing to continue to pass down and develop the traditional culture. Specifically, they hoped to hand down and promote culture through the art design they are good at, such as drawing cartoon characters, illustrations, packaging, logos, and emoji packs.

The traditional culture of the Chinese nation should be passed down, which is also what Chinese people should do (F-01-Q10).

Some learners believed that their cultural attainments (as shown below) had improved. They genuinely believed that producing works based on culture can bring both designers and audiences closer to the culture and improve their cultural attainments through materialized works.

Local culture is a source of inspiration and can make the work more meaningful and attractive (M-02-Q11).

Some students indicated an increase in their personal art and design level (as shown below), which could be attributed to the fact that the integration of interdisciplinary knowledge expanded their creative ideas, enriched them, and made them more determined.

I can use what I have learned to contribute to the transmission of Chinese culture. By doing so, we can not only spread traditional culture but also foster artistic innovation (F-01-Q10).

Some participants were creatively inspired (see below). The Packaging Design curriculum is student-oriented, combines interdisciplinary knowledge and skills, and uses culture as a source of inspiration for design, thereby providing students with constant creative inspiration.

Using a combination of knowledge inspires me to think independently (F-11-Q09).

The respondents believed that they could think comprehensively about each function of packaging because designing packaging requires not only drawing ability but also knowledge of mathematics, science, and engineering, including measuring the size of products to make packages and integrating the characteristics of products to decorate packaging. Considering these factors can enhance the visual impact of their work.

I didn’t have a clue at first about the design process, so I used interdisciplinary knowledge to solve problems (M-08-Q08).

Some learners found that they could complete their work steadily (as shown below). They could acquire considerable knowledge and multiple skills from the Packaging Design curriculum combining C-STEAM, and prepare packages by combining what they had learned with their own personalities, eventually presenting their works in a more complete manner.

We gained an understanding of the culture and then combined our personalities through relevant software, which made the whole package look more complete (M-04-Q08).

Some participants indicated that their artwork recognition improved. Although most students used “Shangshan rice grain” as the main image on the package, the details of the image and the painting style differed considerably. Some designed the top box lid as a cartoon hat with small ears, whereas some replaced the clothing and costumes and integrated culture into their products for display, which was extremely personalized.

Our works became more personalized, for example, the tissue box is in the shape of a lid with small ears on top (F-03-Q08).

Some respondents believed that their precision of packaging design improved, and packaging was accorded effective protection (see below). After taking the course, the students concluded that package dimensions should be measured and calculated using a strict formula, rather than by decreasing or increasing the sizes of some objects. In this manner, the package can fit the size of their products and protect their integrity.

Applying the knowledge of math, I used a ruler to measure the length, width, and height of the package (M-06-Q08).

The students believed that Chinese wisdom continues to be passed down (see below), which is reflected in the fact that the Shangshan ancestors who lived by water made beautiful pottery, stone tools, and other household articles to change their original eating habits, exhibiting their wisdom in a physical manner that continues even to the present day.

The Shangshan people lived by water and created pottery for production and living according to their living environment, turning the kiln into a cooker, which promoted the development and prosperity of human civilization (F-11-Q07).

Some learners felt that their cultural identity and self-confidence in their culture had increased, especially when they produced artworks based on local culture. Furthermore, it strengthened their bond with the culture, deepened their understanding and affirmation of the culture, and subconsciously enhanced their sense of national pride.

When producing various works of art combining local culture, we felt the wisdom of our nation, and our self-confidence in our own culture grew (F-10-Q09).

Some respondents perceived artworks as being full of continuing vitality (as shown below) and believed that traditional local culture provides a solid foundation for modern art design, infusing life into the artwork.

The rich patterns on the pottery are of great significance to our current creations, adding historical value to the work (F-11-Q07).

## 5 Discussion

Focusing on the elements of Shangshan culture, the present study aimed to explore the feelings of the college students completing the art and design C-STEAM course, so that, based on their understanding of China’s outstanding local culture, they could combine the multi-disciplinary knowledge and skills such as science, technology, engineering, art, and math to design and produce themed product packs. According to the results, 72 h of training in packaging design helped these art majors develop innovation, improve their interdisciplinary attainments, practice their ability, boost their cultural self-confidence, and sharpen their sense of patriotism. The findings of Zhan et al. (2020) showed that C-STEAM interdisciplinary education aimed at passing down the excellent local culture not only has great significance for training professionals, but also has a significant impact on the perceptions of cultural context.

Semi-structured interviews were conducted in this qualitative research to explore the learning experience of the students taking the C-STEAM interdisciplinary course and their willingness to acquire the knowledge of C-STEAM and to learn the local culture. Twelve students in the experimental class of the C-STEAM curriculum answered the interview questions in detail. The students’ learning beliefs, actions, and outcomes, and proposed future learning intentions were comprehensively analyzed, providing an explanation of the quantitative study.

Based on the interview texts, this study concludes that positive learning experiences include a deeper interest in studying Chinese culture (in terms of the learning beliefs), the joy of art creation (in terms of the learning actions), improved humanistic attainments, greater ability to explore and think independently, and better problem-solving skills (in terms of the learning outcomes). Based on the statements by Zhan et al. (2020) and Zhan et al. (2022), integrating C-STEAM education can enhance cultural identity and national self-confidence, stimulate students’ innovative ability, broaden their knowledge, sharpen their skills in hands-on experience, and overcome their fear of difficulty. This result is also consistent with comments by Kong (2020) that the design course integrating the STEAM concept can help students develop creativity and inculcate them with the ability to appreciate their culture. Furthermore, they integrated cross-disciplinary study and stimulated their innovation and creative expression in their design works.

This study further concludes that the negative learning experiences included difficulty in choosing packaging materials and structures and poor arithmetic skills, which is consistent with the findings of Zhou (2023), and Zheng and Chen (2023), who highlighted the need to select the design based on products owing to the diversity of packaging design materials and structures that rendered the selection process tedious. Qian and Wang (2021) asserted that because the art and design major in most Chinese higher vocational colleges does not provide mathematics courses, students majoring in art are weaker in mathematics than those majoring in other courses.

All participants in this study expressed their willingness to continue taking the Packaging Design curriculum combining C-STEAM, and to learn more about the C-STEAM course in the future. They expressed their desire to learn more about traditional culture to contribute to the transmission of China’s excellent local culture. They also shared their desire to integrate elements of culture into their works of art in the future. This result is consistent with those of Zhan et al. (2022) and Zhan et al. (2023) who stated that after 3 years of project practice (i.e., from 2016 to 2019), the total number of students interested in participating in interdisciplinary STEAM curriculum increased 15-fold, and the number of students interested in learning about traditional culture increased from 28 to 96%. Fan and Ye (2020) and Ye et al. (2020) reported that after STEAM integration into a fashion design course, students became more willing to invest more time and effort in promoting their art learning performance.

## 6 Conclusion and suggestions

### 6.1 Conclusion

Combining China’s actual conditions, this study designed an art design C-STEAM course integrated with local culture for China’s colleges. The integration of the Shangshan culture into the Packaging Design course proved the importance and significance of adding C-STEAM to the art design course, which helped us understand the specific feelings of students after completing the course, and gain a deeper understanding of the changes in their knowledge and skills and their learning effects. This study, thus, enriches the literature on the development of the art design C-STEAM course in China’s colleges by means of qualitative research.

Based on the BAO model, this study selected four themes from the interviews and analyzed them comprehensively. The results are summarized as follows. First, the learning beliefs of the students taking the Packaging Design course combined with C-STEAM included both positive and negative beliefs. In terms of positive beliefs, a deeper interest in studying Chinese culture was mentioned most frequently. Negative beliefs included a lack of *a priori* knowledge of packaging, a lack of creative ideas at the early stage, and a limited understanding of the meaning of the culture. Second, the learning actions of the students taking the Packaging Design course combined with C-STEAM can be divided into positive and negative aspects and learning suggestions. The positive aspect included the joy of art creation and pleasure in producing works by hand, whereas the negative aspect included the difficulty of choosing packaging materials and structures. In terms of learning suggestions, the interviewed students suggested detailed explanations of each area of knowledge, greater diversity of teaching, study trips, integration of local culture, and increased interaction between teachers and students, providing inspiration for future research and teaching. Third, the learning outcomes of the students taking the Packaging Design course combined with C-STEAM included positive and negative aspects. In terms of the positive aspect, improved humanistic attainments and boosted cultural self-confidence were mentioned most frequently; in terms of the negative aspect, poor arithmetic skills were mentioned most frequently. Fourth, in terms of learning intentions, all interviewed students stated that they could effectively use interdisciplinary knowledge and skills for “personalized” themes. They expressed their willingness to take more C-STEAM courses in the future as well as to learn about traditional culture and produce artworks based on the culture. To summarize, the integration of C-STEAM into the Packaging Design course can help students develop creative expression and art integration abilities, which is consistent with the purpose of the present study.

To accomplish the goal of building a culturally powerful country, the country’s cultural soft power and Chinese cultural influence will be enhanced by cultivating culturally oriented talents, and thereby promoting cultural confidence. This study innovated the role of C-STEAM education in the teaching of art design in a Chinese higher vocational college, effectively applied interdisciplinary knowledge to the teaching process, capitalized on geographical advantages, and analyzed the historical value of the local culture. Efficient use of the local culture can not only ensure its preservation, utilization, inheritance, and development, but can also provide characteristic education in higher vocational institutions and build artistic talent, ultimately achieving a win-win situation. Furthermore, our study provides theoretical support for the academic community from the perspective of art and design, and contributes to the diversity of disciplinary elements.

### 6.2 Contribution

The contributions of this study are as follows: (1) STEM education is seen as the cornerstone of a country’s capacity for innovation and creative development, and is the key to developing and shaping students’ higher-order cognitive abilities and promoting their future career development. To examine specific feelings of students after taking the course and to understand students’ learning effects after course experiments, this study developed a vocational C-STEAM course on art and design with local characteristics, thus enriching research on the development of C-STEAM art and design courses in China’s higher vocational colleges through qualitative research. (2) The study aimed to maximize the in-depth integration of multidisciplinary knowledge of science, technology, engineering, art, mathematics, and local culture. This can encourage students to apply the acquired interdisciplinary knowledge to artistic exploration and creation, as well as accelerate training talent who can satisfy the needs of society. (3) The findings revealed that the students believed that the integration of C-STEAM into the Packaging Design curriculum is an innovative and creative method of teaching that is removed from the previous teaching methods. The course was designed to provide students with time to think, accept their ideas, and respond in a timely manner, thereby contributing to the development of a distinctive art course in China’s higher vocational colleges.

### 6.3 Recommendations

Furthermore, in the subsequent part of the course, based on negative learning experiences in students’ learning beliefs, actions and outcomes, when running the Packaging Design curriculum combining C-STEAM in the future, detailed explanations of science, technology, engineering, art, mathematics, and culture should be provided to students, especially in the areas where they are weak, such as measuring and calculating sizes and assembling packages. More knowledge of traditional local culture should be incorporated, rather than confining the content to the knowledge of the Shangshan culture, to prevent students from merely gaining local cultural knowledge. Moreover, classroom teaching, group teaching, and individual teaching should be provided according to reality. Additionally, teachers must focus on each student’s learning progress in a timely manner and strengthen communication with introverted students and those who are lagging behind.

In terms of the suggestions about students’ learning actions, the course should be improved and extended to continuously optimize the art and design curriculum. For example, a combination of indoor and outdoor teaching activities is necessary. More study trips and field trips to cultural heritage sites and local museums are recommended so that students can understand the unique charm of the culture and broaden their horizons and knowledge. Students’ development should be prioritized, focusing on their participation in class, and multi-dimensional comprehensive evaluation should be conducted from sketches, prototypes, and typesetting to the finished product to avoid one-sided scoring, which would dampen their learning enthusiasm.

Studies on the STEAM curriculum in China have overly imitated European and American courses, which has resulted in a lack of appropriate localization and poor integration with existing courses (Huo et al., 2020; Zhan et al., 2022). Therefore, integrating C-STEAM education into courses related to art and design is highly warranted to encourage students to apply their interdisciplinary knowledge to artistic exploration and creation. Moreover, to effectively develop a curriculum system with local characteristics, teachers should provide more opportunities for students to explore and learn C-STEAM in the future, and integrate multiple elements into their teaching.

### 6.4 Limitations and further study

In this study, semi-structured interviews were used to gain insights into the changes in knowledge and skills of students taking the course, and the learning effects of the course. However, this method could not provide an understanding of the participants’ thoughts, and the results could not be described quantitatively. Therefore, our study can be supplemented with other research methods such as questionnaires in the future to more comprehensively understand students’ feelings about their learning and to gain a more adequate and deeper understanding of the learning effect.

Design innovation poses challenges to experts and art; therefore, how to help art and design students develop creative thinking and ability is an important research theme which needs to be explored continuously. In the future, researchers need to continuously examine the causes of difficulties in producing works and ways to cope with them, the process of design creation, the innovation in teaching and learning modes, teachers’ guidance methods, and so on.

Although the school and major selected for this study are typical of art majors in Chinese higher vocational colleges, the participants were all from the same college. The number of interviewees was limited because of the strict rules enforced by the college regarding the teaching period and the number of students. Therefore, follow-up studies should involve larger samples to explore specific feelings of students majoring in other courses on the theme in various colleges.

C-STEAM is a localized form of STEAM education oriented toward China’s excellent traditional culture (Zhan et al., 2022). Chinese culture is extensive and profound, comprising vivid and unique cultures from distinct ethnic groups and regions (Qian, 2019). In this study, C-STEAM was integrated into the Packaging Design curriculum, including only the Shangshan culture. In the future, more traditional Chinese local cultures can be integrated into the curriculum to create innovative ideas for the development of STEAM education in China. Moreover, the study can offer STEAM courses based on ideological and political education (IP-STEAM) and expand the C-STEAM educational model according to the characteristics of the students’ majors.

## Data availability statement

The raw data supporting the conclusions of this article will be made available by the authors, without undue reservation.

## Ethics statement

The studies involving humans were approved by the Ethics Committee of Dhurakij Pundit University (DPUHREC050/65NA). The study was conducted in accordance with the local legislation and institutional requirements. The participants provided their written informed consent to participate in this study.

## Author contributions

Qian C, and Ye J-H: Concept and design and drafting of the manuscript. Qian C, and Ye J-H: Acquisition of data and analysis. Qian C, Ye J-H and Zheng C: Critical revision of the manuscript. All authors contributed to the article and approved the submitted version.
