# Supplementary material for: Integration of Shangshan culture into the STEAM curriculum and teaching: results of an interview-based study
Source: Front Psychol. 2023 Dec 6;14:1251497. doi: 10.3389/fpsyg.2023.1251497 (PMC10731287; doi:10.3389/fpsyg.2023.1251497)
Supplement: Supplementary file 1 [file Table_1.docx]

Appendix 1

*Curriculum Framework Diagram*

| Course | Content |
| --- | --- |
| 1. Overview of Packaging Design | Characteristics of Shangshan culture (C) |
|  | History and classification of packaging materials (S) |
|  | An overview of process technology (T) |
|  | Principles of packaging structure design (E) |
|  | Packaging style and design elements (A) |
|  | Packaging size and specification (M) |
| 2. Graphic design of packaging | Interpretation of cultural elements of Shangshan culture (C) |
|  | Selection and development of environmentally friendly materials (S) |
|  | Drawing technology (T) |
|  | Characteristics of the internal and external structure of packaging (E) |
|  | Use of text and images (A) |
|  | Measurement and calculation of sizes (M) |
| 3. Process of packaging design | Extraction of cultural elements in the Shangshan culture (C) |
|  | Selection of product-oriented materials (S) |
|  | Process technology (T) |
|  | Process of making packaging structure (E) |
|  | Composition and principles of packaging (A) |
|  | Market research and data summary (M) |
| 4. Integration of culture into packaging production | Use of elements of the Shangshan culture (C) |
|  | Selection of packaging materials with regional characteristics (S) |
|  | Manufacturing technology (T) |
|  | Structural characteristics of a series of gift boxes (E) |
|  | Visual expression of cultural characteristics (A) |
|  | Proportion of finished packaging (M) |

Appendix 2

*Unit I*

| Unit I: Overview of Packaging Design | | |
| --- | --- | --- |
| Extraction of elements in the Shangshan culture (C) | A | Pottery and stone tools, as the most important elements of the Shangshan culture, show the wisdom and ability of the Shangshan people to make production tools in terms of material selection, production, sizing, modeling, and decoration. |
|  | B | Analysis of the geographical environment, climate, and soil structure of the Shangshan site group shows that the Shangshan ancestors had a certain knowledge of geography and could select their residence according to their survival needs. |
|  | C | Rice excavated in the Shangshan site proved that the Shangshan ancestors already knew that rice is edible, that they had initially mastered rice farming techniques and started farming civilization using rice farming as the main mode of production and graduated from constant migration to settling down. |
| Expertise in characteristics and selection of materials (S) | A | Paper is widely used in packaging design because it is cheap, environmentally friendly, easy to print, process, and transport, and suitable for packaging of various products. |
|  | B | Plastic is a symbol of the development of modern packaging design. Because it is cheap, rich in raw materials, and resistant to water, oil, and corrosion, it has become a packaging material that is widely used. |
|  | C | Wood, metal, glass, ceramics, and other packaging materials have a long history and are still being used. |
| Expertise in the application of production techniques and tools (T) | A | Printing process of paper packaging is divided into relief, intaglio, lithography, and screen printing. |
|  | B | Steel wires and knives are used in the mold-cutting and creasing process of cartons and boxes to line up templates to press and cut printed materials into shape. |
|  | C | Transform text or patterns into a convex version and heat up and press aluminum foil paper to prepare gold stamp silver paper. |
|  | D | The double membrane process is categorized into coating and pre-coating, which is surface processing. |
| Expertise in structure and modeling (E) | A | The main structure is constructed according to packed goods and is usually in the shape of a column, cone, disk, or tube. |
|  | B | Locking mouths, self-locking cards, windows, and inter-walls are designed according to the packaging needs of packed goods. |
|  | C | Auxiliary structures, also known as functional structures, are developed to satisfy special needs such as handles, opening, and pour-out. |
| Artistic and aesthetic expertise (A) | A | Different geometric forms are incorporated into the package to make it more spiritual. |
|  | B | Destructive overlays or distortion are added to the packaging to break through original conventions and increase the artistic impact. |
|  | C | The package simulates natural forms to form a bionic shape design, highlighting the fun and vividness, and enhancing the visual impact. |
| Packaging size expertise (M) | A | A large package used for large goods is 530mm * 230 mm * 290 mm-530 mm * 290 mm * 370 mm in size. |
|  | B | A medium package is between 430 mm * 210 mm * 270 mm and 350 mm * 190 mm * 230 mm in size, similar to the size of a computer. |
|  | C | A small package is generally 290 mm * 170 mm * 190 mm in size and is used for decorative items and small ornaments. |
